# HyperCKemia associated with acupuncture: a case report and review of the literature

**DOI:** 10.1186/s12906-021-03484-y

**Published:** 2022-01-28

**Authors:** Xiaochan Tan, Wei Liu, Yuzheng Du, Xianggang Meng, Xuemin Shi

**Affiliations:** 1 First Teaching Hospital of Tianjin University of Traditional Chinese Medicine, Tianjin, China; 2 National Clinical Research Center for Chinese Medicine Acupuncture and Moxibustion, Tianjin, China

**Keywords:** HyperCKemia, Acupuncture, Creatine kinase, Statins, Adverse events

## Abstract

**Background:**

Acupuncture therapy has been widely used as an alternative therapy to treat multiple diseases, such as sequelae of stroke, pain, facial paralysis and so on. In recent years, few adverse events related to acupuncture treatment have been reported, among which hematoma, bleeding and dizziness are the main manifestations. However, to date, there have been no existing cases reported the association between acupuncture therapy and asymptomatic/pauci-symptomatic hyperCKemia.

**Case presentation:**

We report a patient who developed hyperCKemia during 5 sessions of acupuncture at different frequencies. After stopping acupuncture treatment for 1 month, follow-up showed a significant downward trend in serum creatine kinase (sCK). However, after that this patient started to get acupuncture treatment again in order to improve the sequelae of stroke. Meantime, the sCK rose again.

**Conclusion:**

HyperCKemia may associated with acupuncture therapy. All kinds of adverse events of acupuncture should be recorded comprehensively and objectively so as to improve the safety standard system of acupuncture therapy.

## Background

Acupuncture therapy, which originated in China, is one of the most popular complementary and alternative therapies in many countries at present [1]. Many clinical studies have demonstrated the effectiveness of the treatment in many diseases such as sequelae of stroke, lumbar disc protrusion, periarthritis of shoulder and primary hypertension [2, 3]. In terms of safety, although they are not common, some adverse events (AEs)-such as pneumothorax, central nervous system injury, syncope, infection, bleeding, allergy-do occur during acupuncture treatment [4, 5]. However, according to our search, there has been no case yet reported that acupuncture is associated with asymptomatic/pauci-symptomatic hyperCKemia, which is defined as serum creatine kinase (sCK) > 1.5 times the upper limit of normal(ULN) [6], namely 504 U/L.

## Case report

**Fig. 1:**
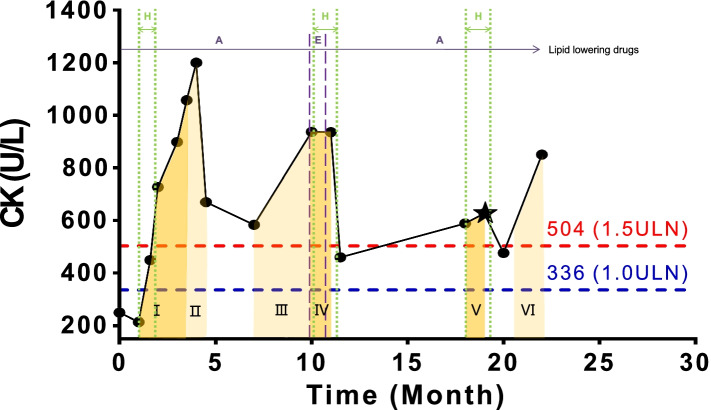
The relationship between sCK and acupuncture treatment and the use of lipid-lowering drugs. Description: ★:August 2020. A: Atorvastatin (duration of use: Jan. 29th 2019 to Nov. 11th 2019 and Dec. 3th 2019 to Nov. 6th 2020). E: Ezetimibe (duration of use: Nov. 12th 2019 to Dec. 2nd 2019). H: Herbal medicine (duration of use: Feb. 13th 2019 to March. 5th 2019,Nov. 13th 2019 to Dec. 3th 2019 and Jul. 7th 2020 to Aug. 18th 2020). Phase I/IV/V: acupuncture treatment, 5 times / week. Phase II/III/VI: acupuncture treatment, 3 times / week

We reported a case of a 58-year-old male patient with abnormal sCK who came to our clinic in August 2020. On January 29th 2019, he noticed the abnormity of sCK for the first time. Since then, he has regularly reviewed sCK and found abnormal, discontinuous fluctuations in it. He had previous medical history including (1) an acute cerebral stem infarction occurred in January 2019. Symptomatic treatment left sequelae including mild aphasia, dysphagia, and numbness and weakness in the right side of the body (2) acute pancreatitis which has recovered after treatment (3) a left acetabular fracture resulting from car accident trauma occurred 18 years ago and left leg weakness after surgery (4) hypertension (5) hyperlipidemia (6) diabetes. After fully understanding the patient’s treatment history and medical history of sCK from January 29th 2019 to November 6th 2020, we draw Fig. 1 and classified the patient’s treatment into two categories. One of the treatments is western medicine. For the last 2 years, he has been receiving daily standard treatment, including metformin (500 mg/d), amlodipine besylate (2.5 mg/d), atorvastatin (20 mg/d) and aspirin (100 mg/d). Except from November 12th 2019 to December 2nd 2019, his doctor advised him to replace atorvastatin(20 mg/d) by ezetimibe (10 mg/d) as the doctor considered that elevated sCK might be associated with the use of statins. After that, he changed back to use atorvastatin, since there was no significant change in sCK as well as the elevation of triglycerides and fibrinogen during the drug change. The other category of the treatments is traditional Chinese medicine. The patient mentioned that in order to improve the sequelae of stroke, he had received three acupuncture treatments at different hospitals 3–5 times a week in the past 18 months, and had taken three courses of herbal medicine. All acupuncture treatment is purely manual operation, without the use of electroacupuncture, the choice is also the traditional acupoints that often used in the treatment of stroke, such as Neiguan (PC6), Shuigou (DU26), Sanyinjiao (SP6), Baihui (DU20), Yifeng (SJ17), Jianyu (LI15), Taichong (LR3). The composition of herbal medicine in the three courses was slightly different, among which the drugs repeatedly used were *Pinellia ternata*, Magnolia officinalis, Poria cohoe, Perilla leaf and cassia twig, which were used to invigorating spleen to eliminate dampness and warming yang for activating qi-flowing.

In addition, we reviewed the results of multiple physical examinations taken by the patient in the past 2 years to analyze the possible causes of his elevated sCK based on the Guidelines for the diagnosis of CK elevation were published by the European Neurological Union in 2010. During his medical examination in 2019, his electrocardiogram, B-type natriuretic peptide (BNP) and creatine kinase MB (CK-MB) isoenzyme tests were normal, and echocardiography did not indicate morphological changes of the heart, which could exclude the occurrence of heart failure and myocarditis. Although he felt weakness in both legs, the neurological examination was normal: the tendon reflexes draw out normally, the limb muscle strength was grade 5 (according to the Lovett strength test), muscle tension and volume were normal (according to the Modified Ashworth Scale), and there was no edema or color change (paleness and cyanosis). Compared with a year and a half ago, his symptoms such as limb dysfunction after cerebral infarction also improved significantly after acupuncture treatment. Unfortunately, the patient refused to have an electromyogram (EMG) or a muscle biopsy to further explore the cause.

**Fig. 2:**
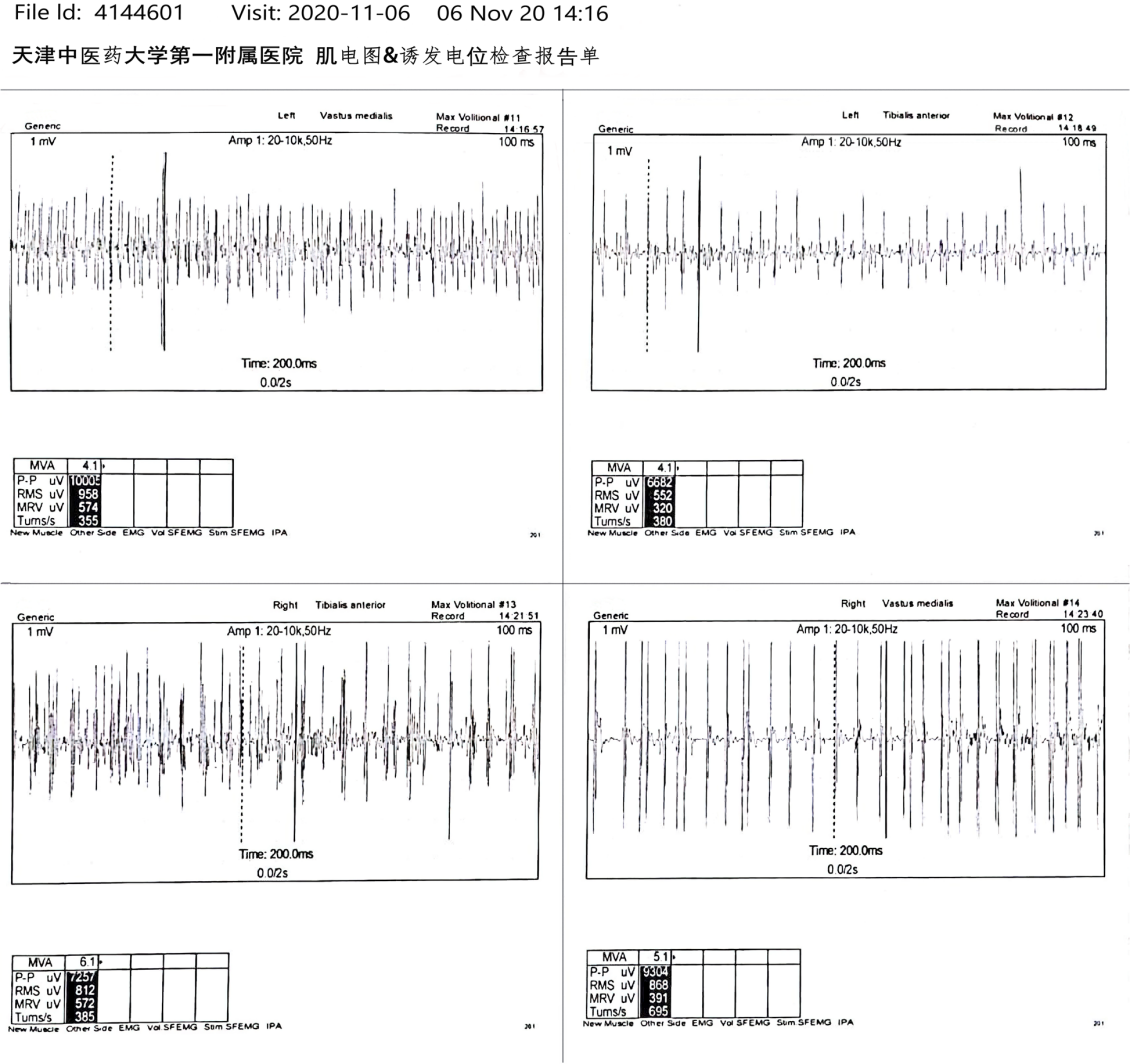
EMG results of extremities

In summary, by sorting out the patient’s medical history, we did not find that he had the diseases that could raise the level of sCK mentioned in the guidelines. But combined with Fig. 1, it can be seen that the increase of sCK was highly related to acupuncture treatment, and the rate of increase was associated with the frequency of acupuncture. In view of this situation, we suggest the patient to stop current acupuncture treatment since August 12, 2020, maintain a good lifestyle, prohibit strenuous exercise, and review sCK regularly. Subsequently, an obvious phenomenon shows that the sCK value drops significantly to 476.1 U / L on 11th September 2020 (after phase V in Fig. 1). But what is puzzling is that there was a sharp upward trend in November (phase VI in Fig. 1). During detailed questioning, the patient admitted that he accepted acupuncture treatment (3 times/week) again since late October 2020 in order to improving the swallowing disorder. In addition, during the follow-up, the patient agreed to accept an EMG examination. And the results showed that no myogenic lesion was found in both lower extremities, however, neurogenic lesion could not be excluded (Fig. 2). Therefore, we advised the patient to stop acupuncture, monitor sCK value, and seek other therapies such as functional training.

A consultation team (consisting of specialists from our hospital’s acupuncture, neurology, cardiology and endocrinology departments) was involved in the diagnosis of the case. Based on the patient’s medical history and laboratory results, this case was diagnosed as hyperCKemia. The cause and/or complications of this diagnosis are thought to be:

1. Acupuncture induction, caused by: The fluctuation of sCK in patient is highly correlated with the time of acupuncture treatment, and the dose-CK response curve of acupuncture also shows high intensity, especially phase III, V, VI (Fig. 1). After stopping acupuncture treatment for a period of time, there were significant upward trends in phase III, V and VI when he resumed acupuncture treatment and had no changes in medication. Especially, we can observe a significant decline between phase V and VI which may be caused by the removal of acupuncture intervention. The only variable factor in all the above phases is whether acupuncture treatment was applied. Therefore, the correlation between acupuncture treatment and the changes of sCK can be clearly observed in Fig. 1. According to World Health Organization Collaboration Center for International Drug Monitoring (The Uppsala Monitoring Centre, WHO-UMC) [7] and Naranjo method [8], the causal relationship between acupuncture treatment and hyperCKemia was assessed as “probable/ likely”.

2. The onset of cerebral infarction may cause the elevation of sCK. In this case, the patient’s first increase of sCK (phase I:12th February,2019 ~ 25th April,2019) occurs next to the acute phase of cerebral infarction (29th January,2019 ~ 11th February,2019). Some reports [9–12] have shown that many patients with acute cerebral infarction will have an increase in sCK, and the sCK levels correlate with the extent of cerebral, and most patients reach the peak in 4–6 days. This change may be related to many factors, among which catecholamine theory is the most important. It should be noted, however, that there is no consensus or guidance on this point.

3. Side effects of lipid-lowering drugs cannot be ruled out. Since the stroke, this patient has been taking the lipid-lowering drugs atorvastatin and ezetimibe, and the increase of sCK with myalgia is one of the known adverse effects of lipid-lowering drugs, particularly statins [13, 14]. However, the patient only felt myasthenia without myalgia, and the neurological examination was normal. Experts of the diagnosis team considered that the symptoms of myasthenia were not statin related myopathy, but might be the sequelae of stroke or diabetic peripheral neuropathy. Moreover, in an assessment recommendation updated by Robert in 2014 [15], it was pointed out that the clinical significance between increased sCK without muscle symptoms and statins use was uncertain.
4. Herbal medicine’s side effects are also possible. However, when searching relevant literature, we found that only one study showed that Angelica sinensis and kudzu root induced the increase of CK in high-fat diet mice [16], while more studies showed that the correct use of herbal medicine would reduce the activity of CK or its subtype isozyme in myocardial ischemia animal models [17–19]. In this case, the patient did have an elevation of sCK in the three courses of taking herbal medicine, and since different herbal medicines are often used in combination, it is difficult to determine whether the ingredients that may cause the elevation of sCK will be produced. However, as can be seen from Fig. 1, sCK continued to increase after the patient stopped taking herbal medicine in phase I, and the increase of sCK in phase III and VI was not related to herbal medicine.

Importantly, among the four factors mentioned above, only acupuncture showed significant correlation with the four abnormal increases in patient’s sCK (Including phase I, III, V, VI in Fig. 1), while the other factors did not. The occurrence of early cerebral infarction may influence the elevation of sCK, but the relationship between late cerebral infarction and sCK is unclear. In addition, the fluctuation of sCK value is not completely correlated with lipid-lowering drugs and Chinese medicine courses, so we believe that these two are not the main influencing factors.

## Discussion and conclusion

Acupuncture has long been considered as a relatively safe treatment, most of the adverse events which associated with it were mild and self-limiting [20]. A cumulative review [21] including a total of 715 adverse events showed that the risk of serious adverse events occurring in association with acupuncture is very low, which was estimated to be 0.05 per 10,000 treatments, and 0.55 per 10,000 individual patients.

The increase of sCK is often found to be related to the use of statins. Severe adverse reactions to other drugs, such as malignant syndrome caused by antipsychotics, may also be manifested as elevated sCK. However, acupuncture has rarely been reported to be associated with it. Only one case [22] reported that severe rhabdomyolysis, which was diagnosed basing on clinical (muscle weakness) and laboratory findings (elevated sCK, serum glutamic oxaloacetic transaminase, lactate dehydrogenase, phosphorus, uric acid and hemoglobinuria), occurred after acupuncture sessions. On the contrary, many studies have shown that acupuncture may have the potential function to reduce sCK. A recent meta-analysis [23] showed that acupuncture treatment after intense exercise may benefit alleviating delayed-onset muscle soreness (DOMS) and decreasing the serum level of sCK (SMD: − 0.91, 95%CI: − 1.27 to− 0.56, *P* < 0.001, I^2^ = 30%). Although the small number and low quality of including trials may affect the results, we can see that acupuncture may have a completely opposite effect on the level of serum sCK under different circumstances. The existing evidence is too limited to infer whether this discrepancy is caused by individual differences or other reasons, further research is needed to explore its mechanism.

We proposed a case which suggested that there may be a correlation between acupuncture and the increase of sCK. Although it is hard to completely rule out the influence of drugsand other potential diseases, his long-term hyperCKemia is most likely induced by acupuncture from the time curve of sCK changes of the patient. sCK is a muscle enzyme which is related to generating energy crucial for muscle function [24]. The elevation of sCK may occur when there is muscle injury or sarcolemmal disruption [25] . Although we cannot define the precise mechanism that caused the rise of sCK, after excluding other possibilities, we educed that the inserted acupuncture needles may cause muscle injury which might lead to the increasing of sCK. Therefore, we suggest that the history of acupuncture treatment should be asked when clinicians treating patients with single elevated sCK. In addition, acupuncture should also be regarded as one of the possible reasons for the increase of sCK in the treatment of patients with cerebrovascular disease. Otherwise, patients may be unnecessarily denied treatment with statins.

Although acupuncture has advantages on improving the sequelae of stroke, we still should attach great importance to its limitation and side effect. Since WHO formulated the acupuncture and moxibustion operation standard in 1999 [26], many countries including United States and Japan have also formulated the corresponding acupuncture and moxibustion safety standard [27, 28] according to their national conditions which ensures the safety of acupuncture and moxibustion treatment to a certain extent. However, it is worth noting that the security specification has not formed a complete system. This case highlights the possibility of acupuncture leading to hyperCKemia. We suggest that sCK should be included in the evaluation of acupuncture safety. It is also important to note the etiological diagnosis of hyperCKemia. After excluding the possibility of other physiological, neurological and drug-related factors, the influence of acupuncture should be considered, so as to better evaluate and arrange the treatment plan for patients.

## Data Availability

All data generated or analysed during this study are included in this published article.

## Abbreviations

AEs: adverse events
sCK: serum creatine kinase
ULN: upper limit of normal
BNP: B-type natriuretic peptide
CK-MB: creatine kinase MB isoenzyme
WHO-UMC: World Health Organization Collaboration Center for International Drug Monitoring (The Uppsala Monitoring Centre)
DOMS: delayed-onset muscle soreness
